# From decentralization to commonization of HIV healthcare resources: keys to reduction in health disparity and equitable distribution of health services in Nigeria

**DOI:** 10.11604/pamj.2016.24.266.6286

**Published:** 2016-07-21

**Authors:** Obinna Ositadimma Oleribe, Olabisi Abiodun Oladipo, Iheaka Paul Ezieme, Mary Margaret Elizabeth Crossey, Simon David Taylor-Robinson

**Affiliations:** 1 Excellence &Friends Management Care Centre (EFMC), Abuja, Nigeria; 2 Hepatology Unit, Imperial College London, 10th Floor, QEQM Building, St Mary’s Hospital Campus, South Wharf Road, W2 1NY, London, United Kingdom; 3 Department of Medicine, Jos University Teaching Hospital, 2, Murtala Mohammed Way, Jos, Plateau State, Nigeria

**Keywords:** Decentralization, Commonization, HIV/AIDS, Health disparity, Nigeria

## Abstract

Access to quality care is essential for improved health outcomes. Decentralization improves access to healthcare services at lower levels of care, but it does not dismantle structural, funding and programming restrictions to access, resulting in inequity and inequality in population health. Unlike decentralization, Commonization Model of care reduces health inequalities and inequity, dismantles structural, funding and other program related obstacles to population health. Excellence and Friends Management Care Center (EFMC) using Commonization Model (CM), fully integrated HIV services into core health services in 121 supported facilities. This initiative improved access to care, treatment, support services, reduced stigmatization/discrimination, and improved uptake of HTC. We call on governments to adequately finance CM for health systems restructuring towards better health outcomes.

## Essay

### Decentralization and commonization model

**Figure 1:**
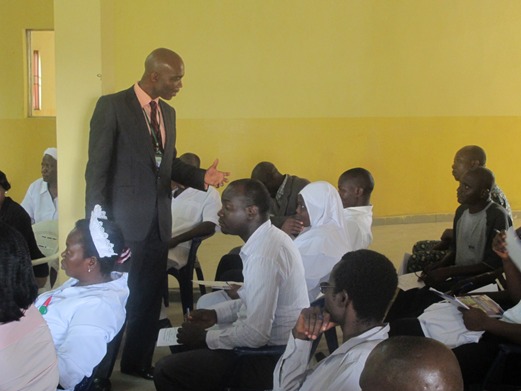
On-site training at General Hospital, Abaji, Abuja, Nigeria 2013

**Figure 2:**
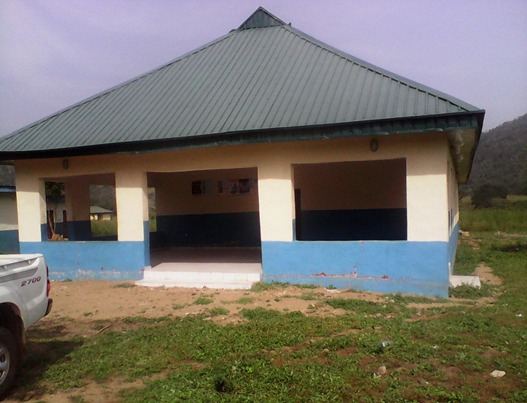
Kujekwa primary health center centre in Abuja, Nigeria - 2014

**Figure 3:**
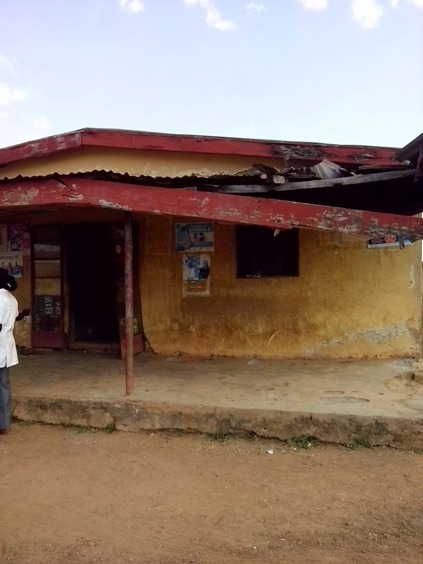
Primary health care center in a hard-to-reach area with very poor infrastructures – but supported by EFMC to provide HIV services

Universal health coverage (UHC), basically a health financing mechanism founded on pooling of funds and designed to provide health care coverage for a country’s entire population, is increasingly seen as a silver-bullet solution to healthcare needs in low and middle-income countries [1]. The central tenet of UHC is a desire that all people receive health services they need without suffering financial hardship by paying for them [2]. UHC is programmed to protect people against the high costs of healthcare that can push people into debt and poverty, whether in the public or private domain [3]. However, payment for health services is only important when health services are available and geographically accessible. To achieve geographical accessibility of services, nations have had to decentralize their health care system, allowing lower levels of care (mainly primary health care) to provide services that were previously seen at the tertiary and secondary levels (Figure 1). Decentralization usually involves devolution of power, delegation and de-concentration of services from tertiary centers to primary health care centers (PHCs), leading to various degrees of decision space [4]. It offers patients and health care clients a choice between different levels and types of health institutions that vary in terms of their levels of sophistication, areas over which they have jurisdiction, and the spectrum of services available within the establishment. It is a highly popular concept as it is linked with the emergence and globalization of primary health care, and is a key element in PHC policies [5]. Decentralization with local control of resources is an alternative to the traditional vertical disease program approach for priority interventions [6]. The gains of decentralization have led to devolution and de-concentration of key health issues, such as HIV/AIDS from the federal to the local communities, and from tertiary centers to primary centers in various countries (Figure 2). However, mere decentralization is not enough. Beyond decentralization, services must be commonized to achieve equitable distribution of health care services as well as universal health coverage [7]. According to Oleribe et al., in 2014 [7], Commonization Model (CM) has three main arms: **(1) Integration of services into the fabric of the hospital:** this allows HIV patients to receive care, treatment and support services along with other patients in the center. With CM, there are no special clinics, clinic days, health workers, laboratory, wards, pharmacy or even operating theatres for HIV patients. HIV-positive patients are seen alongside HIV-negative individuals and they are attended to in all sections of the hospital. **(2) Decentralization and devolution of treatment to all levels of care:** CM decentralizes services from tertiary to secondary, primary health centers and health posts; with prior training and empowerment to provide specific levels of care to HIV-infected individuals and other health care seekers. In extreme cases, CM empowers traditional birth attendants, maternity homes and patent medicine vendor’s provides pecified levels of care including HIV testing and counseling, condom distribution and referrals, with some serving as ART drug pick-up sites. **(3) Provision of HIV services in hard-to-reach communities and villages, where there are no HIV services:** majority of hard-to-reach communities and villages do not have ‘standard’ health facilities. CM requires that services be provided for the people in these communities in a culturally acceptable way and place, which sometimes may be in a community health worker’s house, community centers or the chief’s palace (Figure 3). Over a 36 months period, (September 2011-October 2014), Excellence and Friends Management Care Centre (EFMC) achieved various levels of Commonization in HIV services in Abuja-FCT, Nasarawa and Imo States in Nigeria. This article discusses the need for complete Commonization of health services, especially HIV/AIDS services. It also discusses the rationale behind EFMC’s Commonization Model and its benefits.

### The need for commonizationof HIV services in Nigeria

Nigeria has the second highest burden of HIV in the world, after South Africa [8]. The first case of HIV/AIDS infection in Nigeria was discovered in a 13 year old girl in 1986 and since then, the epidemic has spread and today Nigeria has generalized epidemic in all the 36 + 1 States. In 1991, the Ante-natal clinic (ANC) sentinel survey placed the adult HIV prevalence at 1.2%. The HIV prevalence grew exponentially from 3.8% in 1993, 5.4% in 1999 to 5.8% in 2001; but declined to 5.0% in 2003, 4.4% in 2005 and 4.1% in 2010 [9]. The National HIV/AIDS and Reproductive Health Survey (NARHS) carried out in 2012 put the national adult HIV prevalence at 3.4%, which also showed a slight decrease from 3.6% in 2007 [10]. According to the National Agency for the Control of AIDS (NACA), as of 2013, Nigeria has about 3,229,757 adults and children living with HIV with about 220,394 new infections. Sexual transmission still accounts for about 80% of all new HIV cases in Nigeria; while mother-to-child transmission, shared needle usage, transfusion of infected blood and blood products account for other modes of transmission. The Agency also estimated that a total of 1,476,741 required anti-retroviral drugs in 2013, out of which 639,397 received treatment, resulting in a total of 210,031 deaths from AIDS-related causes in the same year[8]. The adult HIV prevalence was highest among those aged 35 to 39 (4.4%), and lowest among those aged 15 to 19 (2.9%), and generally higher among females than males [10]. The same report also revealed that HIV prevalence was highest in the South-South zone of the country with a prevalence of 5.5% and lowest in the South-East zone with prevalence estimated at 1.8%. As at 2013, only 6,675,000 people were tested for HIV [11] and this represents about 4% of the total population of Nigeria while, only 17% of HIV positive women received anti-retroviral (ARV) drugs for prevention of mother-to-child transmission (PMTCT) [12]. Since 2004, when treatment of HIV/AIDS started effectively in Nigeria, it has been largely donor dependent. In 2007, 85.4% of all HIV expenditure was derived from external sources and this increased to 92.35% in 2008 [13]. Nigeria is the third largest beneficiary of the United States President’s Emergency Program for AIDS Relief (PEPFAR) fund and has received over US$2 billion since 2004, through a number of channeling organizations such as the Center for Disease Control and Prevention (CDC). Funds are provided through implementing partners to support scale-up of HIV testing, treatment, care, prevention of mother-to-child transmission, and capacity building of healthcare workers [14]. Using these support funds, sites were assessed, health workers trained, commodities supplied and facilities activated to provide HIV services. Because of the funding requirements, these treatment sites supported by foreign donors provide HIV services through vertical or stand-alone programs with specialized personnel, separate laboratories, specific clinic days and separate budgets, resulting in high cost of programming, lack of sustainability and increased stigma and discrimination among HIV-infected and affected persons. However, financial support to Nigeria’s HIV program is gradually reducing as a result of the current status of Nigeria as the largest economy in Africa [15] and the development of the President’s Comprehensive Response Plan (PCRP) by the Government of Nigeria [16]. The Nigerian President’s Comprehensive Response Plan (PCRP) was developed to serve as a platform through which increased government contribution to the national response to the HIV/AIDS epidemic will be channeled. It aims to bridge the funding resource gap for the HIV program in Nigeria, improve access to HIV/AIDS services and accelerate implementation of key interventions, such as HIV testing, initiation of eligible persons on ART, elimination of mother-to-child transmission (eMTCT), increase provision of combination prevention services for key populations and the activation of new PMTCT and ART service delivery points across the country over a 2 year period [16].

### The commonization process model assumption

EFMC is a specialist public health management care center and provides sustainable public health services with emphasis on HIV/AIDS, malaria and reproductive health. EFMC received funding from the US government in 2011 to run a unique project called “Reaching All with Care and Support Services in HIV/AIDS” (REACH). The project continued after the first year as “AIDS Relief Interventions with Systems Enhancement” (ARISE) through a sub-granting arrangement. To commonize HIV services in Nigeria, we made the following assumptions; Assumption 1- Health care workers are willing to learn and treat HIV-infected persons if given the opportunity. Assumption 2- Individuals living in hard-to-reach areas are willing to use health care facilities closest to them if quality services are provided in a culturally acceptable manner. Assumption 3- Health care workers working in PHCs are willing to take on additional services if there is adequate support for the services. Assumption 4- Funding limitation is not the only obstacle to universal health coverage. Although these assumptions will be tested in future works, they guided the entire Commonization process.

### Implementation of commonization model

**Figure 4:**
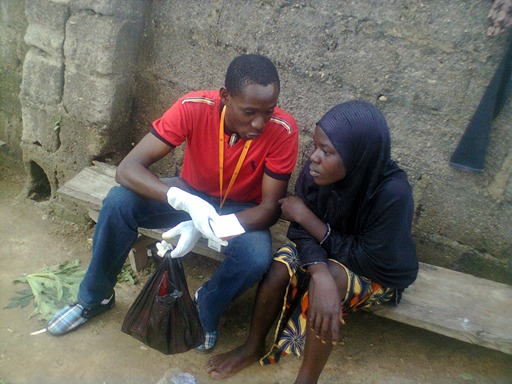
House-to-house HIV testing and counseling in Abuja, Nigeria

**Figure 5:**
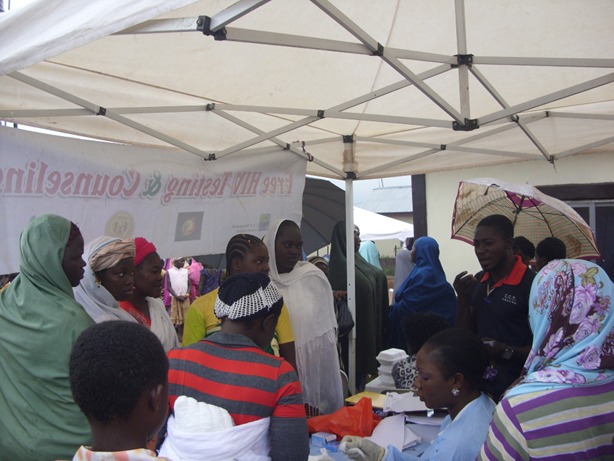
Medical outreach with free HIV testing and counseling in a community by EFMC and partners

The CM fully integrated HIV services into core health services. This model dismantled parallel HIV programs existing in sites which were experienced in HIV service provision. EFMC also refused to fund parallel structures in newly engaged activated sites, dismantling special task force for HIV within the supported facility. Special HIV clinic/specific HIV clinic days, special HIV laboratory, special HIV pharmacy and special HIV personnel were integrated into hospital systems. Detailed facility assessment, and on-site orientation on healthcare workers on all aspects of HIV care using standardized national guidelines were done. In all orientations and trainings, EFMC emphasized the similarity of HIV infection to other common chronic diseases, and encouraged all healthcare workers to see HIV-infected persons as individuals with chronic diseases, and should not be perceived as a “special” disease. Following assessment, tailored training, and activation of the sites, EFMC provided site staff with on-the-job mentoring, data collection and reporting tools, drugs, consumables, and laboratory equipment. EFMC staff also supported the facilities to adopt and take ownership of the HIV program, patient management and data collection. These were designed to ensure sustained quality service delivery and continuation of services when funding stops. Provider-initiated HIV testing and counseling (PITC), recommended by the World Health Organization (WHO) in 2007, was strongly advocated for, as hospital patients were found to have higher positive rates than the general community. This practice also capitalized on all patients and their contacts within the medical system. Every hospital patient’s visit was seen as an opportunity for HIV testing, diagnosis and linkage to care. This increased testing participation, and identified HIV-infected individuals at earlier disease stages [17]. Also, HIV testing was provided at multiple points at each supported healthcare center. These additional sites where located in the emergency units, wards, OPD clinics, immunization clinics, STI clinics, etc. This made access to testing and counseling services easier as people who needed HIV tests did them on the spot, without having to move long distances to central laboratories, that commonly resulted in default [7]. Outside the hospital environment, mobile testing, house-to-house (H2H) and shop-to-shop (S2S) testing at communities were used to provide HIV testing where people lived and work (Figure 4). This demystified the infection. CM ensured that persons who conventionally would not visit health care centers were reached and tested for HIV. Testing people where they live and work also empowered people with the knowledge of their HIV status, thus validated the assumption that people would access services if brought closer to them in culturally acceptable forms. In addition to the above strategies, EFMC engaged primary healthcare centers (PHC) and private medical vendors (PMV). Since only about 17% of the population sought healthcare in public general and teaching hospitals, involvement of the PMVs and PHCs facilitated access and reduced inequity in the distribution of health care services[18], especially in hard-to-reach zones. This also further demystified HIV and HIV care as abstract and inaccessible to the common man. Significant improvement was noticed in uptake and prompt enrolment of more patients into ART (Figure 5). Loss to follow up reduced (especially among pregnant women), despite greater geographic HIV care coverage [19].

### Outcome of commonization model of delivery

A total of 121 (101 Primary, 12 Secondary and 8 Private) health care facilities were assessed, trained and activated to provide HIV services in three states-Imo, Nasarawa and Abuja-FCT between October 2011 and March 2013 using the Commonization Model. These included facilities in remote areas with minimal health infrastructures and difficult terrain, increasing cost of activation and monitoring. CM provided services to rural dwellers, who were previously unserved despite infrastructural challenges. EFMC focus on these centers facilitated refurbishing of a number of them by government and other interested organizations and provision of adequate human resources. EFMC also funded the upgrade and refurbishing of 24 health facilities in Imo State between 2013 and 2014. New structures, such as a hospital laboratory, were constructed in a few supported centers. Relevant equipment, work stations, shelves, tables and chairs to ensure continuity of services in these centers were provided. Infrastructure upgrade was limited to a few centers because of funding limitations, but where they took place, they raised the morale of the healthcare workers, boosted their commitment and ensured their full participation in the Commonization process. They also made the facilities attractive to the community and their surrounding neighbors, thus increasing uptake of services within these facilities.

### Current and potential benefits of commonization model to HIV programs

**Figure 6:**
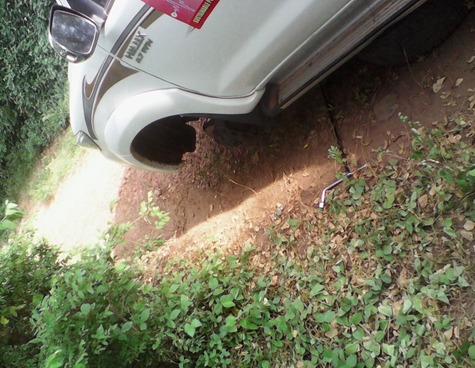
Operational vehicle damaged by very bad road network to one of the hard-to-reach sites

Commonization of HIV services eliminated the alienation of the rural population, who constitute greater proportion of the target population, and reduced the higher risk of uncontrolled transmission of HIV commonly seen in rural areas. The recent population surveys in Nigeria revealed a rising prevalence of HIV in rural communities from 3.5% in 2007 [20] to 3.6% in 2012 [10]. Drivers of this rising prevalence include low health literacy level, ignorance of protective measures, inequitable access to mass media, and poor access to services. Thus, there are often high unsafe sexual networks within rural areas, with one unidentified infected person posing serious threat to the general population making contact tracing and case identification very important in these scenarios. Commonization also reduces cultural practices that increase risk of infection, minimizes stigma and discrimination; and other untoward effects of HIV burden of care such as poverty, malnutrition and family disintegration. Commonization also increases condom awareness and use in rural areas. EFMC’s decision to make services available in these remote areas brought services within the reach of the rural dwellers, empowering them with the knowledge of HIV and their HIV statuses, and facilitated the setting up of measures to prevent new infections. It also guided the community on steps to take when diagnosed with the HIV infection, provided HIV care, treatment and support services where they work and live. In addition, out of pocket expenditures reduced significantly as they do not have to travel long distances to secondary or tertiary health centers to access basic care such as HTC, condoms, ARV prophylaxis and treatment; adherence and compliance are better (Figure 6). This has therefore, reduced the client load at the secondary and tertiary health facilities. Making HIV care services available at the primary healthcare facilities in remote areas resulted in greater acceptability of services, increased referrals and enrolments into HIV care and improved patient retention, compared to limiting services to specialized hospitals [21]. This confirmed the assumptions that people are willing to use services when provided in a culturally acceptable form. EFMC improved the capacity of healthcare workers in various aspects of the HIV program-HIV testing and counseling, provision of prophylaxis to HIV-infected pregnant women, adherence counseling, drug dispensing, infant feeding counseling, provision of prophylaxis to HIV-exposed infants, dried blood spot (DBS) sample collection and referral of reactive clients to treatment sites; leading to better quality of care to HIV-infected persons.

### Sustainability of HIV programs in low and middle income countries

**Figure 7:**
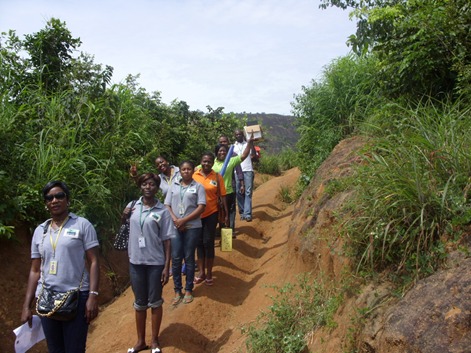
EFMC staff walking to a hard-to-reach destination following the breakdown of the operational vehicle

Developed countries of the world are able to keep their HIV prevalence at very low levels through effective public health measures that include adequate screening for disease detection in populations, early commencement of ARVs for all eligible patients, lifestyle modification as well as treatment of other ailments that cause morbidity and mortality. These practices are grossly lacking in Nigeria. In order to achieve sustainability in the HIV program in Nigeria, there is an urgent need for the government to increase the budgetary allocation to health from 6.4% (budget for 2014) to the 15% recommended in the Abuja declaration of 2001[22]. Beyond allocation, the government should also ensure technical efficiency as they must ensure both allocative and technical efficiency in the release of these funds for programming and health service activities. In addition, resources should be effectively utilized in the infrastructural and manpower development of the health sector, especially in the PHCs. Physical infrastructure upgrade of all existing PHCs (regardless of location), posting of relevant community healthcare workers trained in provision of basic HIV services and provision of necessary work tools and consumables is the first step towards universal health coverage and Commonization as this will improve access to health services where people work and live. Strong referral linkages to community support groups and treatment centers should be in place so as to ensure continuum of care. At the treatment centers, task shifting and task sharing can be implemented to distribute the workload among healthcare workers (Figure 7). The National Health Insurance Scheme (NHIS) is an opportunity for all Nigerian citizens to be provided with basic health care without costly out-of-pocket expenditure. This scheme should be made available and accessible to all Nigerians and not just a proportion of the populace. With the recent signing of the National Health Bill into law that aims to protect and prioritize the rights of Nigerians to get the basic minimum package of healthcare, quality and comprehensive primary healthcare should be extended to Nigerians living in hard-to-reach rural communities [23].

### Conclusion and recommendations

The HIV program has evolved over the years. The programs have been largely supported by international agencies and foreign partners. However, with the current emerging ‘donor fatigue’, it is time to source funding for the HIV program from within in order to make basic healthcare services available and accessible for every citizen. Universal Access can be achieved by making these services available as close to the grassroots as possible. The Primary Healthcare Centers are the most basic source of health services in Nigeria but have been given the least attention by successive governments, in terms of funding for infrastructure, manpower provision and development. EFMC provided services where other organizations were unlikely to serve and thus provided HIV/AIDS services to people living in hard-to-reach communities. It is a daunting task that costs more in man-hours, travel and vehicle maintenance, consumable and communication. However, the smiles on the faces of people touched, the lives saved and health restored were all worth the efforts. If the national and global targets for testing and enrolment into HIV care are to be met, limiting services to urban based facilities will not achieve the goal. The government and practitioners should buy into the concept of Commonization, make health services available to all people where they live and work, and integrate HIV services into the fabrics of the health system. This will improve the health of the people. This work validates all the assumptions made and revealed that health care workers are willing to learn and treat HIV-infected persons if given the opportunity; individuals living in hard-to-reach areas are willing to use health care facilities closest to them if quality services are provided in a culturally acceptable manner; health care workers working in PHCs are willing to take-on additional services if there is adequate support for the services; funding limitation is not the only obstacle to universal health coverage. However, these assumptions need to be tested in a more structured research and in other communities before findings could be generalized. Commonization of health services will demystify the notion that HIV care can only be provided for those living in the urban areas by a select group of health personnel in special treatment centers. However, Commonization should go beyond just HIV services, care and treatment; and be applied to all segments of health services. This will strengthen the health system, improve health indices, increase productivity and help achieve current health focused millennium development goals (MDGs) and proposed sustainable development goals (SDGs). To achieve this, government and health practitioners must overcome the challenges of access by constructing good roads, improve health infrastructures which currently are very weak, especially at the primary health care level and encourage health workers to work in rural communities through improved welfare packages and incentives. Implementing partners should also work at scaling out services to hard-to-reach areas, refurbish community-based facilities, train community-based health workers, and provide basic equipment to replace non-functional ones. This will have cost implications and partners should prioritize based on available resources [24]. If not built into the project, this will result in implementation challenges. Also, as some of these health facilities are located in very remote areas with bad or inexistent access roads, driving on these roads to provide support to the HIV/AIDS services as well as conduct regular supervisory/monitoring visits at these sites could cause severe damages to operational vehicles, as experienced by EFMC staff on several occasions. Be prepared to make sacrifices.
